# A modified vertical pressure bandage to prevent pharyngocutaneous fistula after total laryngectomy

**DOI:** 10.1016/j.bjorl.2024.101537

**Published:** 2025-01-03

**Authors:** Xuwei Duan, Jian Xu, Xueqin Liu, Duoping Wang, Biaoyou Chen

**Affiliations:** a Guangxi Medical University Cancer Hospital, Department of Head and neck Surgery, Nanning, China; b Guangxi Medical University Cancer Hospital, Department of Hospital Quality Control Management, Nanning, China

**Keywords:** Pharyngocutaneous fistula, Modified vertical pressure bandage, Total laryngectomy, Retrospective study

## Abstract

**Objective:**

Pharyngocutaneous Fistula (PCF) is one of the most common and challenging postoperative complications after Total Laryngectomy (TLE). This study aimed to evaluate the impact of the modified Vertical Pressure Bandage (VPB) on the occurrence of Pharyngocutaneous Fistula (PCF) after Total Laryngectomy (TLE) for Head and Neck (H&N) cancer.

**Methods:**

This retrospective study included patients with H&N cancer who underwent TLE at our hospital between January 2010 and January 2021. The patients were grouped according to whether modified VPB was used or not after surgery. The primary outcome was the occurrence of PCF. The secondary outcomes were the interval from operation to PCF, the healing time of PCF, and the length of hospital stay.

**Results:**

This study included 133 patients: 82 (aged 57.93 ± 10.18 years, 80 males) in the VPB group and 51 (aged 54.98 ± 9.22 years, 51 males) in the conventional group. Patients who received the modified VPB had a significantly lower occurrence of PCF compared with those did not (9.8% vs. 33.3%, *p* = 0.001). In addition, the patients in the VPB group had similar interval from the operation to the PCF (*p* = 0.374) and healing time of PCF (*p* = 0.256) but a significantly shorter length of hospital stay (*p* < 0.001) compared with those in conventional group. Moreover, multivariable logistic regression analysis showed that, after adjusting for age, preoperative radiotherapy, postoperative hematocrit < 35%, VPB (RR = 0.165, 95% CI 0.057‒0.474, *p* = 0.001) was an independent protective factor for PCF.

**Conclusion:**

The modified VPB might reduce the occurrence of PCF after TLE for H&N cancers.

**Level of evidence:**

Level 4.

## Highlights

• Modified VPB lowers PCF post-TLE for H&N cancers.
• Modified VPB: lower PCF, shorter hospital stay.
• Multivariable analysis confirms modified VPB independently protects against PCF.
• Modified VPB is potential to be an alternative process.

## Introduction

Total Laryngectomy (TLE) is a surgical procedure conventionally used to treat advanced-stage laryngeal and hypopharyngeal cancer.^1^, ^2^ The procedure involves high morbidity and has numerous complications.^1^, ^3^, ^4^ Pharyngocutaneous Fistula (PCF) is one of the most common and challenging postoperative complications after TLE.^1^, ^3^, ^4^ PCF can lead to delayed wound healing, prolonged hospitalization,^5^ an increased risk for the development of distant metastases,^6^ and a higher readmission risk within 30 days.^3^ The reported occurrence of PCF varies widely from 1.3% to 34%, depending on the selected patient cohort.^7^, ^8^, ^9^, ^10^, ^11^, ^12^, ^13^

There are many risk factors associated with PCF, such as tumor site,^13^ preoperative radiotherapy,^14^ low skeletal muscle mass,^15^ heavy smoking,^16^ low level of hemoglobin,^17^ positive surgical margins,^13^, ^18^ neck dissection,^18^ preoperative albumin,^5^ wound classification of 3 and 4 vs. 1–2, preoperative transfusion of >4 units of packed red blood cells,^12^ Neutrophil-to-Lymphocyte Ratio (NLR) > 2.5,^19^ and postoperative hemoglobin.^13^ Many of these risk factors are intrinsic to the disease or the patient and cannot be modified. Still, even though not all patients develop a PCF, it is difficult to manage once it occurs.

Few studies are focusing on the prevention of PCF. Based on long-term practice experience, the authors modified the Vertical Pressure Bandage (VPB) to prevent PCF. The VPB of the neck is widely used after parotidectomy to prevent parotid leakage. Its stress points are in the parotid gland, submental, and overhead, and there is no compression on the carotid sinus. In the clinical practice on the prevention of PCF, it was observed that if the force point of the VPB is moved to the position between the original hyoid bone and the hypopharynx and bandage the hypopharynx after TLE, PCF can be significantly reduced. Therefore, this study aimed to evaluate the impact of the modified VPB on the occurrence of PCF after TLE for Head and Neck (H&N) cancers.

## Methods

### Study design and patients

This retrospective study included patients with H&N cancer who underwent total laryngectomy in the H&N Surgery Department of our hospital between January 2010 and January 2021. The inclusion criteria were 1) Patients who underwent TLE for H&N cancer, 2) Followed up for at least 30 days. Patients with incomplete data were excluded. This retrospective study was approved by the Ethical Committee of our Hospital. The requirement for informed patient consent was waived by the committee because of the retrospective nature of the study. We have followed STROBE reporting guideline.

### Data collection and definition

The patients were grouped according to whether the modified VPB (Supplementary Materials) was used or not after surgery. The procedures of TLE and modified VPD are shown as Supplementary Materials. The demographic and pre-, intra-, and postoperative clinical data were extracted from the medical charts, including age, sex, current smoker, current drinker, diabetes mellitus, heart disease, chronic obstructive pulmonary disease, hypertension, Body Mass Index (BMI), Tumor Node Metastasis (TNM) stage, tumor site, preoperative hematocrit < 35%, preoperative hypoproteinemia, preoperative White Blood Cell (WBC) abnormality, preoperative radiotherapy, preoperative chemotherapy, preoperative tracheostomy, number of surgeons, duration of surgery, neck lymph node dissection, perioperative blood transfusion, doctors for dressing change, postoperative hematocrit < 35%, postoperative hypoproteinemia, and postoperative WBC abnormality.

### Outcomes

The primary outcome was the occurrence of PCF. The secondary outcomes were the interval from operation to PCF, the healing time of PCF, and the length of hospital stay.

### Statistical analysis

All statistical analyses were performed using SPSS 19.0 (IBM Corp., Armonk, NY, USA). The continuous variables were analyzed for normal distribution using the Kolmogorov-Smirnov test. Those with a normal distribution were presented as means ± Standard Deviation (SD) and analyzed using Student’s *t*-test; otherwise, they were presented as medians (interquartile ranges) and analyzed using the Mann-Whitney *U*-test. The categorical data were presented as n (%) and analyzed using the Chi-Square test or Fisher’s exact test. A forward-LR stepwise multiple logistic regression model was used to identify the risk factors for PCF. In the univariable analysis, variables with *p* < 0.05 were included in a multivariable logistic regression analysis to explore the association between the vertical pressure bandages and PCF. Two-sided *p*-values <0.05 were considered statistically significant.

## Results

A total of 133 patients were included: 82 (80 males, aged 57.93 ± 10.18 years) received the modified VPB (VPB group), and 51 (51 males, aged 54.98 ± 9.22 years) received conventional dressing change (conventional group). The rates of preoperative hematocrit < 35% (*p* = 0.034), preoperative hypoproteinemia (*p* = 0.007), preoperative tracheostomy (*p* = 0.004), postoperative hypoproteinemia (*p* = 0.001), and postoperative WBC abnormality (*p* = 0.003) were significantly different between the VPB and the conventional groups (Table 1).

**Table 1:** Demographic and clinical characteristics.

Variable	All (n = 133)	Conventional group (n = 51)	VPB group (n = 82)	*p*
Age (mean ± SD)	56.80 ± 9.763	54.98 ± 9.22	57.93 ± 10.18	0.091
Sex, n (%)				0.524
Male	131	51 (39%)	80 (61%)	
Female	2	0	2	
Current smoker, n (%)	102	40	62	0.834
Current drinker, n (%)	87	36	51	0.354
Diabetes mellitus, n (%)	5	2	3	>0.999
Heart disease, n (%)	14	4	10	0.565
Chronic obstructive pulmonary disease, n (%)	5	1	4	0.649
Hypertension, n (%)	27	6	21	0.075
Body mass index, n (%)				0.999
Low	26	10	16	
Normal	78	30	48	
Obesity	29	11	18	
TNM stage, n (%)				0.851
3	51	21	30	
4a	56	19	37	
4b	23	10	13	
4c	3	1	2	
Tumor site, n (%)				0.462
Laryngeal	90	33	57	
Hypopharynx	30	12	18	
Thyroid	5	2	3	
Vocal Cords	5	3	2	
Others	3	2	1	
Pre-operative hematocrit < 35%, n (%)	46	12	34	0.034
Preoperative hypoproteinemia, n (%)	22	3	19	0.007
Preoperative WBC abnormality, n (%)	22	12	10	0.195
Preoperative radiotherapy, n (%)	17	9	8	0.285
Preoperative chemotherapy, n (%)	26	6	20	0.077
Preoperative tracheostomy, n (%)	55	13	42	0.004
Nº of surgeon, n (%)				0.003
1	81	29	52	
2	9	7	2	
3	21	12	9	
4	5	1	4	
5	17	2	15	
Duration of surgery, median (range)	240.0 (205.0–325.0)	260 (210.0–348.0)	235.0 (194.5–302.5)	0.079
Neck lymph node dissection, n (%)	130	49	81	0.558
Perioperative blood transfusion, n (%)	13	8	5	0.080
Nº of doctor for dressing change, n (%)				0.067
1	39	14	25	
2	34	12	22	
3	34	20	14	
4	14	3	11	
5	12	2	10	
Postoperative hematocrit < 35%, n (%)	88	29	59	0.074
Postoperative hypoproteinemia, n (%)	76	20	56	0.001
Postoperative WBC abnormality, n (%)	60	15	45	0.003

VPB, Vertical Pressure Bandage; WBC, White Blood Cells.

Finally, 25 patients developed a PCF, with 8/82 (9.76%) cases in the VPB group and 17/51 (33.33%) in the conventional group (*p* = 0.001). In addition, the patients in the VPB group had similar interval from the operation to the PCF (*p* = 0.374) and healing time of PCF (*p* = 0.256) but a significantly shorter length of hospital stay (*p* < 0.001) compared with the conventional group (Table 2).

**Table 2:** Outcomes.

Outcomes	Conventional group (n = 51)	VPB group (n = 82)	*p*
Primary outcome			
PCF			0.001
Yes	17 (33.33)	8 (9.76)	
No	34 (66.67)	74 (90.24)	
Secondary outcomes			
Days from operation to PCF	12.0 (9.0–13.50)	10.0 (6.50–14.00)	0.374
Healing time of PCF	30.0 (20.0–50.0)	43.50 (27.50–97.50)	0.256
Length of hospital stay	57.0 (36.0–85.0)	35.0 (29.75–48.50)	<0.001

VPB, Vertical Pressure Bandage; PCF, Pharyngocutaneous Fistula.

The univariable logistic regression analysis showed that VPB (RR = 0.216, 95% CI: 0.085‒0.550, *p* = 0.001) potentially reduced the occurrence of PCF, but the relationship could be confounded by age (*p* = 0.022), preoperative radiotherapy (*p* = 0.003), postoperative hematocrit < 35% (*p* = 0.017). The multivariable logistic regression analysis showed that after adjusting for age, preoperative radiotherapy, postoperative hematocrit < 35%, VPB (RR = 0.165, 95% CI 0.057‒0.474, *p* = 0.001) were independent factors for PCF (Table 3).

**Table 3:** Univariable and multivariable logistic regression analysis for the primary outcome.

Variables	Univariable analysis		Multivariable analysis	
	RR/OR, 95% CI	*p*	RR/OR, 95% CI	*p*
VPB	0.216, 0.085‒0.550	0.001	0.165, 0.057‒0.474	0.001
Conventional	Ref	‒	Ref	‒
Age	0.945, 0.901‒0.992	0.022	0.951, 0.903−1.001	0.054
Current smoker	1.268, 0.433‒3.715	0.665		
Current drinker	1.453, 0.558‒3.786	1.453		
Diabetes mellitus	1.083, 0.116‒10.134	0.944		
Heart disease	0.304, 0.038‒2.444	0.263		
Chronic obstructive pulmonary disease	3.043, 0.481‒19.264	0.237		
Hypertension	0.289, 0.064‒1.310	0.107		
Body mass index	0.360, 0.094‒1.381	0.136		
TNM stage	2.687, 0.217‒33.278	0.441		
Tumor site	2.200, 0.493‒9.811	0.301		
Preoperative hematocrit < 35	0.867, 0.343‒2.194	0.763		
Preoperative hypoproteinemia	2.411, 0.861‒6.749	0.094		
Preoperative WBC abnormality	2.000, 0.634‒6.311	0.237		
Preoperative radiotherapy	5.176, 1.754‒15.281	0.003	3.293, 0.951‒11.407	0.060
Preoperative chemotherapy	1.024, 0.344‒3.045	0.966		
Preoperative tracheostomy	1.143, 0.475‒2.750	0.766		
Neck lymph node dissection	0.453, 0.039‒5.201	0.525		
No. of surgeon	1.375, 0.435‒4.343	0.587		
Duration of surgery	1.003, 0.999‒1.007	0.103		
Perioperative blood transfusion	2.095, 0.589‒7.449	0.253		
Nº of doctor for dressing change	2.560, 0.574‒11.408	0.218		
Postoperative hematocrit < 35%	4.667, 1.315‒16.564	0.017		
Postoperative hypoproteinemia	1.156, 0.477‒2.803	0.749	7.615, 1.877‒30.888	0.004
Postoperative WBC abnormality	1.135, 0.474‒2.714	0.777		

VPB, Vertical Pressure bandage; OR, Odds Ratio; CI, Confidence Interval; WBC, White Blood Cells.

## Discussion

The present study showed that patients who received the modified VPB had a significantly lower occurrence of PCF compared with those did not. Multivariable logistic regression analysis showed that, after adjusting for age, preoperative radiotherapy, postoperative hematocrit < 35%, and postoperative hypoproteinemia, VPB were independent factors for PCF. These findings suggested that the modified VPB might reduce the occurrence of PCF after TLE for H&N cancers and potential to be an alternative process.

In the present study, the univariable logistic regression analyses showed that age, preoperative radiotherapy, postoperative hematocrit < 35%, and postoperative hypoproteinemia were associated with the occurrence of PCF. These results are globally supported by the literature.^12^, ^13^, ^14^, ^15^, ^17^, ^18^ Benson et al.^7^ also observed that postoperative anemia was associated with PCF, possibly due to decreased blood oxygen-carrying capacity and tissue hypoxia. Still, these factors are intrinsic to the patients and are non-modifiable (i.e., age and preoperative radiotherapy) or can be controlled but without indication, whether the control interventions will influence the development of PCF (i.e., postoperative hematocrit < 35% and postoperative hypoproteinemia). Therefore, active methods to prevent the development of the PCF are necessary.

At the authors’ hospital, the dressing method was modified to decrease the risk of PCF. It is widely accepted that the necessary condition for wound healing is a well-aligned wound, with no physiological cavity, low suture tension, no foreign body, no postoperative infection, no serious postoperative malnutrition, and minimal movement of the lips of the wound.^20^, ^21^ How to eliminate physiological cavities and limit the movements of the wound lips after TLE are challenging problems. Umezawa et al.^22^ found that cigarette-type continuous negative pressure drainage devices can promote the repair of PCF in patients with postoperative PCF of laryngeal, hypopharyngeal, and esophageal cancer. Inatomi et al.^23^ also observed that negative pressure wound therapy is an effective and minimally invasive treatment for postoperative fistula. Their study demonstrated that continuous negative pressure could force the mucosal tissues together, encourage granulation and adhesion of the transferred soft tissue, reduce dead space, and promote fluid discharge. Still, negative pressure dressings require devices that are not necessarily readily available in all hospitals, especially in developing countries. This study showed that the modified VPB was independently associated with a lower occurrence of PCF after TLE, probably through continuous external pressure and elimination of the dead space, preventing the formation of the PCF.

Vertical pressure bandages were originally used for pressure bandaging after parotid gland operation to prevent parotid gland leakage.^24^ The stress points of the bandage were under the chin, parotid gland, and overhead. The method was improved to compress the hypopharynx. The stress points of the bandage were hypopharyngeal and overhead. The bandage produced continuous backward and upward pressure on the hypopharyngeal (Fig. S1). The external compression could eliminate the physiological dead space and limit the movements of the hypopharyngeal wound, leading to more effective wound healing. The method significantly reduced the occurrence of PCF.

The VPB reduced the occurrence rate of PCF, which shortened the length of hospital stay and reduced the cost of hospitalization. Still, the method has some weaknesses. Due to the continuous change of the patient’s head position during sleep, the bandage of vertical compression bandage is easy to loosen. Although there were no significant differences in PCF in the different doctors who used the same method, 25 patients who underwent vertical pressure bandages in the author’s management had no PCF. According to the author’s experience, the bandages were wrapped using a standard procedure. During dressing change, the bandage of the horizontal line should be wrapped around the apex of the occipital protrusion, which can prevent the bandage from moving up and down. In addition, the bandage on the vertical line should cover the highest point on the top of the skull to prevent the bandage from shifting back and forth. The duration of the pressure bandage should be about 15 days. Future studies need to investigate the impact of different bandage times on the occurrence of PCF.

Previous studies reported the use of flaps for the prevention of PCF.^8^, ^25^ Although these methods appear effective, they involve additional procedures, costs, and donor site morbidity and complications. The cost-effectiveness of such procedures compared with the VPB should be examined.

This study had limitations. It was a single-center study with a limited number of patients. The retrospective nature of the study limited the data to those available in the charts, patients’ data, such as methods of pharyngeal closures, radiation doses, flaps used, and etc., were not available. The dressing in the conventional group was variable and dependent upon the physicians.

## Conclusion

In conclusion, the VPB was independently associated with a reduction of the PCF occurrence rate after TLE in patients with H&N cancer. The use of that simple and convenient method should be further examined.

## CRediT authorship contribution statement

Xuwei Duan and Jian Xu carried out the studies, participated in collecting data, and drafted the manuscript. Xueqin Liu and Duoping Wang performed the statistical analysis and participated in its design. Biaoyou Chen participated in acquisition, analysis, or interpretation of data and draft the manuscript. All authors read and approved the final manuscript.

## Consent for publication

Not applicable.

## Funding

None.

## Data availability

All data generated or analyzed during this study are included in this article and supplementary information files.

## Declaration of competing interest

The authors declare no have conflicts of interest.
