# Supplementary material for: A modified vertical pressure bandage to prevent pharyngocutaneous fistula after total laryngectomy
Source: Braz J Otorhinolaryngol. 2025 Jan 3;91(2):101537. doi: 10.1016/j.bjorl.2024.101537 (PMC11753971; doi:10.1016/j.bjorl.2024.101537)

**BJORL-D-24-00132_Supplementary Material**

**Supplementary Materials**

All patients were treated surgically by the same medical team and nursed according to the standard care protocol. The intraoperative resection range of TLE includes hyoid bone, laryngeal body, partial hypopharynx, and anterior epiglottic space. Pharyngeal reconstruction was performed in three layers: mucosal, fascia, and muscular. If there were few hypopharyngeal mucosal defects, a suture was performed directly. We use absorbable sutures instead of mechanical staplers to suture wounds. The suture process is shown in Figure S1. If there were many hypopharyngeal mucosal defects and the suture tension was high, repairing the hypopharyngeal cavity with a pedicled skin flap or a free skin flap was necessary. All patients were administered appropriate prophylactic antibiotics following the national guidelines published by the Ministry of Health of the People’s Republic of China. Cefazolin was used prophylactically, and the antibiotic use began half 1h before the operation and continued 24‒48h after the operation. All patients were fed a gastric tube.

There were two methods of dressing change after the operation. In the conventional group, the neck was covered with the conventional dressing method. The neck wound was covered with gauze, and the hypopharynx was not bandaged. In the VPB group, 6‒8 gauzes were padded on the skin of the hypopharyngeal surface. Then, the hypopharyngeal was wrapped with vertical pressure bandages. The trend of the bandage was divided into vertical lines and horizontal lines. The horizontal line passed through the forehead, the upper margin of bilateral auricles, and the occipital eminence. The vertical line passed through the highest point of the head, the posterior edge of the auricle on the affected side, the hypopharynx, and the front of the healthy side of the ear. When bandaging, the bandage was wrapped for two turns by the horizontal line, then wrapped for about six turns by the vertical line, the bandage by the horizontal line for one turn, and the bandage was finally fixed. The hypopharyngeal was wrapped by vertical pressure bandages for about 15 days and until the patient could eat normally for 3 days. A typical case is shown in Figure S2.

**Figure S1** The suture process of after total laryngectomy. (A) Wound after total laryngectomy. (B) Inverted suturing of the pharyngeal mucosa. (C) Intermittent suturing of submucosal layer. (D) Intermittent suturing of anterior cervical muscle. (E) Intermittent suturing of the skin.


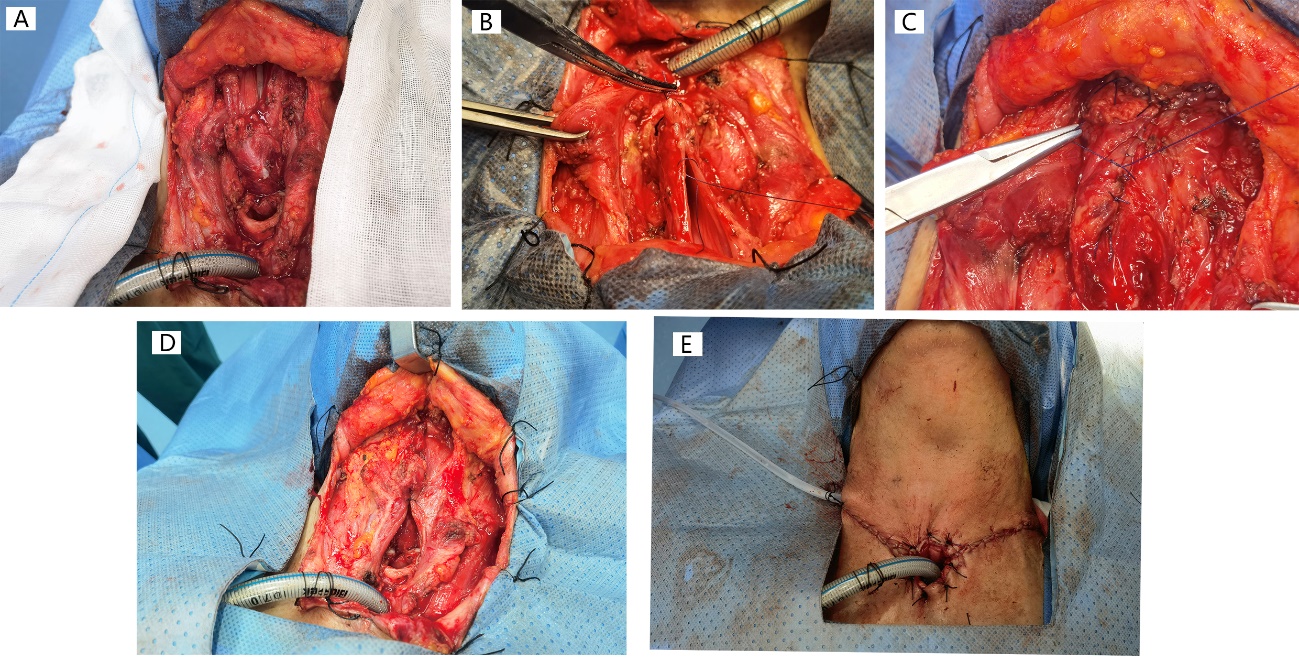


**Figure S2** A 60-year-old male patient received a Vertical Pressure Bandage (VPB) after a total laryngectomy. (A) Right (affected) side. In the sagittal position of the hypopharynx, the resultant force F can be decomposed into an upward component F2 and a backward component F1. (B) Left (healthy) side. In the sagittal position of the hypopharynx, the resultant force F can be decomposed into an upward component F2 and a backward component F1. (C) Front view. In the coronal position of the hypopharynx, the left bandage has a backward upward force F (L), and the right bandage has a backward upward force F (R). Both force (L) and force (R) have a vertical upward component F2 and an upward and backward component F1. Therefore, there is a pressing force from both sides to the midline between force (L) and Force^®^, squeezing the hypopharyngeal tissue upward, backward, and inward. (D) Back view. (E) Front view. After loosening the bandage, there is an obvious indentation in the hypopharynx.


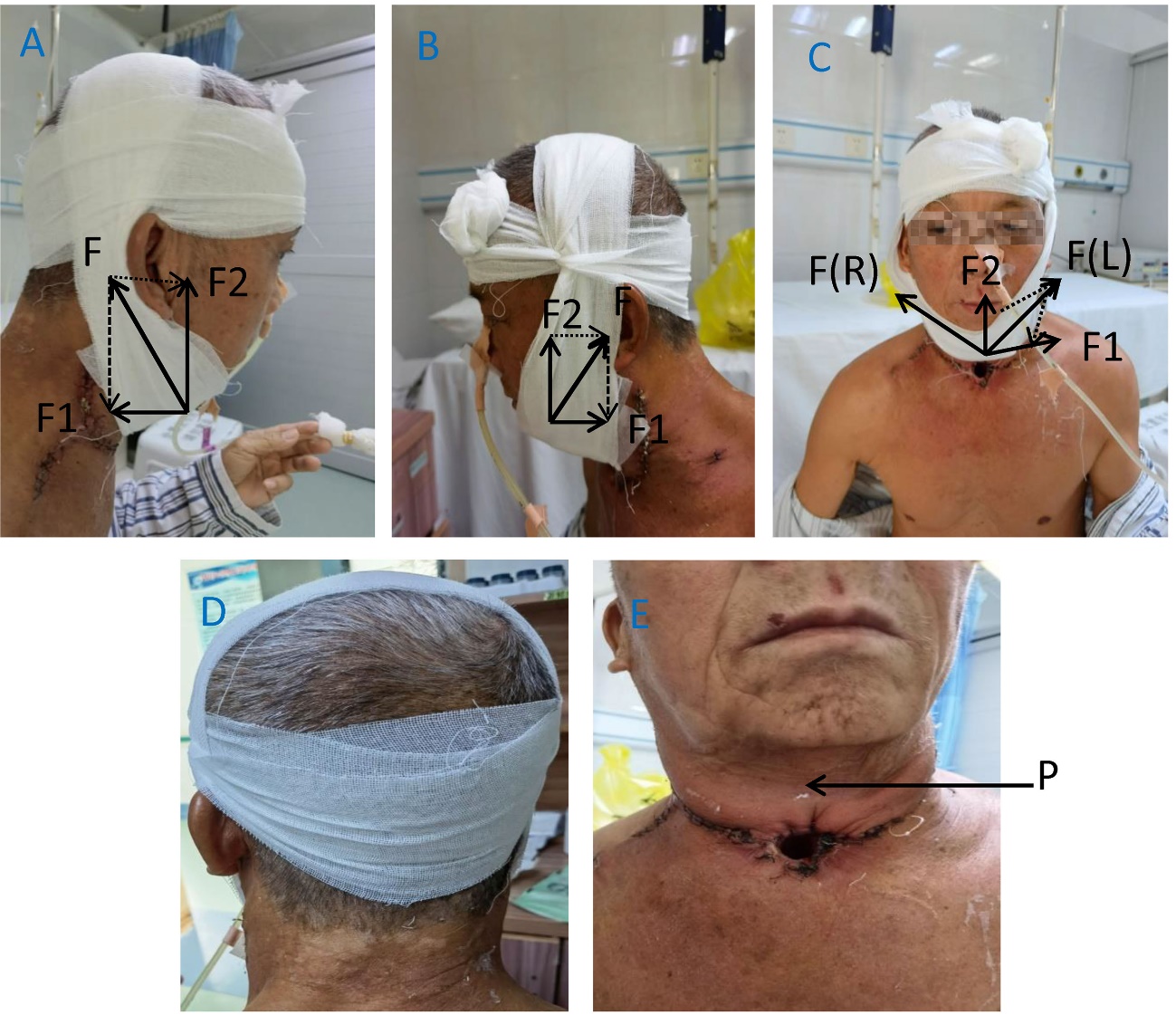

Supplement: Supplementary file 1 [file mmc1.docx]
